# Whole transcriptome analysis in offspring whose fathers were exposed to a developmental insult: a novel avian model

**DOI:** 10.1038/s41598-023-43593-x

**Published:** 2023-10-01

**Authors:** Issam Rimawi, Sunny Yanai, Gadi Turgeman, Joseph Yanai

**Affiliations:** 1 The Ross Laboratory for Studies in Neural Birth Defects, Department of Medical Neurobiology, Institute for Medical Research – Israel-Canada, The Hebrew University-Hadassah Medical School, P.O. Box 12272, 91120 Jerusalem, Israel; 2 Department of Genetics, The Institute of Life Sciences, The Hebrew University of Jerusalem, Jerusalem, Israel; 3 Department of Molecular Biology, Ariel University, Ariel, Israel; 4 Department of Pharmacology and Cancer Biology, Duke University Medical Center, Durham, NC 27710 USA

**Keywords:** Developmental biology, Genetics, Molecular biology, Neuroscience

## Abstract

Although the effects of paternal exposure to insults on the offspring received limited attention in the past, it is currently gaining interest especially after understanding the mechanisms which may mediate such exposure effects. In the current study, the well-controlled avian model (*Fayoumi*) was utilized to investigate the effects of paternal exposure to the developmental insult, chlorpyrifos on the offspring’s gene expression via mRNA and small RNA sequencing. Numerous mRNA gene expression changes were detected in the offspring after paternal exposure to the developmental insult, especially in genes related to neurogenesis, learning and memory. qPCR analysis of several genes, that were significantly changed in mRNA sequencing, confirmed the results obtained in mRNA sequencing. On the other hand, small RNA sequencing did not identify significant microRNA genes expression changes in the offspring after paternal exposure to the developmental insult. The effects of the paternal exposure were more pronounced in the female offspring compared to the male offspring. The results identified expression alterations in major genes (some of which were pertinent to the functional changes observed in other forms of early developmental exposure) after paternal insult exposure and provided a direction for future studies involving the most affected genes.

## Introduction

The behavioral and molecular deficits observed in the offspring after prenatal/pre-hatch exposure to insults such as chemicals, drugs and pesticides has been well documented^1–4^. Reversal of neurobehavioral defects after exposure to different teratogens was applicable after understanding the mechanisms mediating such effects^5–7^.

Prenatal exposure to insults has and still is extensively investigated, whereas preconception insult exposure received minimal consideration^8^. Consequences of parental preconception insult exposure on the offspring were first investigated in 1915^9^. However, only recently did this area of research started to gain attention, especially paternal exposure to insults (for review, see^8^). This is mainly caused by enhanced knowledge of the mechanisms that may mediate deficits in inheritance including histone modifications, sperm RNA transmission, and DNA methylation (for review, see^10^).

As an archetypal developmental insult, we chose chlorpyrifos^11,12^. The choice of this substance was not related to its role as a toxicant but because it is a well-known and extensively studied neurobehavioral teratogen by numerous groups including our own^3,12–20^.

Prenatal exposure to neuroteratogens including chlorpyrifos, heroin and alcohol is frequently associated with gene expression alterations. Mice and human fetuses exposed to chlorpyrifos show neurobehavioral changes (deficits in exploration and anxiety in mice and in cognition and language in humans) along with modifications in gene expression^21,22^. Several studies have demonstrated neurobehavioral changes (deficits in learning, memory, dendritic branch length, cholinergic and dopaminergic innervations) in the offspring exposed to heroin prenatally^3,23^. Those changes were also associated with modifications in gene expression^24^. Behavioral deficits observed with fetal alcohol syndrome after prenatal exposure to alcohol could be caused by disturbances in the regular control of reprogramming and gene expression in the embryos^25^.

Previous paternal investigations, mostly employing rodent models, studied the deleterious effects of a variety of insults exposure (including therapeutics, ionizing radiation, lifestyle influences, etc.) on the offspring and observed behavioral, biochemical, and/or molecular alterations (for review, see^8^). However, only few studies considered investigating gene expression alterations in the avian model after paternal insult exposure^26^, and none considered analyzing the whole transcriptome using next generation sequencing techniques. Consequently, it became an obvious next step to study alterations in gene expression in the offspring paternally exposed to insults using mRNA and small RNA sequencing in the novel avian model.

Unlike most avian based neurobehavioral teratology investigations which use layer or broiler species^27^, the current investigation was performed on the rare strain, *Fayoumi* which is typically prone to severe environment (for review, see^28^) and is known to be less vulnerable to poultry infections^29^. This makes it ideal for the current research where a vigorous strain is required that could survive severe teratogen exposure.

Whole-transcriptome analysis of differential gene expression can be provided through RNA sequencing technique. Greater coverage and higher resolution of the transcriptome’s dynamic nature are considered the main advantages of RNA sequencing over the previous Sanger sequencing and microarray-based methods (for review, see^30^). Currently, RNA sequencing methods can be used in different aspects of RNA biology, including RNA structure, translation and single-cell gene expression^31^.

As was shown in our previous investigations^3,32^, the expression of neurogenesis and neurotransmission genes was affected in the offspring after pre-hatch exposure to the developmental insults, chlorpyrifos. Other gene expression and epigenetic modifications and defects in signaling cascades were observed after pre-hatch/prenatal exposure to chlorpyrifos in human^21^ and animal models^33–36^. Studies applying rodent models showed that offspring exposed to neuroteratogens parentally (preconception exposure) may also demonstrate gene expression^37,38^ and epigenetic^39^ modifications and signaling cascades defects^40,41^.

In addition to our previous studies that demonstrated neurogenesis, neurotransmission, learning and memory alterations in the offspring after pre-hatch exposure to the developmental insult^3,32^; our recent study^26^, performed on the paternal and maternal preconception models (using qPCR analysis), indicated neurogenesis, neurotransmission and epigenetic genes expression changes in the offspring after paternal exposure. Taken together, these observations provided the rationale to our hypothesis that paternal exposure to the developmental insult would have extensive effects on the offspring’s gene expression, especially genes related to neurogenesis, neurotransmission, learning and memory. Additionally, the identification of other significantly altered genes in the female and male offspring using RNA sequencing techniques will contribute to further understand the mechanisms that mediate alterations in those offspring and pave the way for further mechanistic-functional studies.

Consequently, in the current study, whole-transcriptome analysis was performed to provide a comprehensive evaluation of gene expression changes in the offspring paternally exposed to chlorpyrifos. Male *Fayoumi* chickens were administered chlorpyrifos for two weeks before mating with untreated females. mRNA and small RNA sequencing analyses were performed on brain samples collected from the embryos prior to hatching (incubation day 20). RNA sequencing results were then validated using real-time qPCR analysis which was performed on several genes.

## Methods

Chickens were cared for in accordance with the office of laboratory animal welfare (OLAW) of the national institutes of health (NIH)^42^. All procedures have been approved by the Institutional Animal Care and Use Committee (IACUC)—Hebrew University of Jerusalem and were performed in accordance with the accepted guidelines and regulations (Approval Code: AG-19-15635; Date of approval: 8/12/2018). All experiments were performed in accordance with ARRIVE guidelines.

### Chicken housing

Female and male *Fayoumi* chicken were maintained in our animal facilities under standard laboratory conditions as previously described^26^. Chickens were 7–10 months old and were divided into separate groups, where group members (of females and males) were replaced frequently to increase genetic variability, while it also carried the limitation of tracking the eggs back to their original parents. To enable the replacement, parents were taken from a flock of the paternal group. There were always 2 males and 5 females in each cage for each replication.

### Chlorpyrifos administration

**Figure 1:**
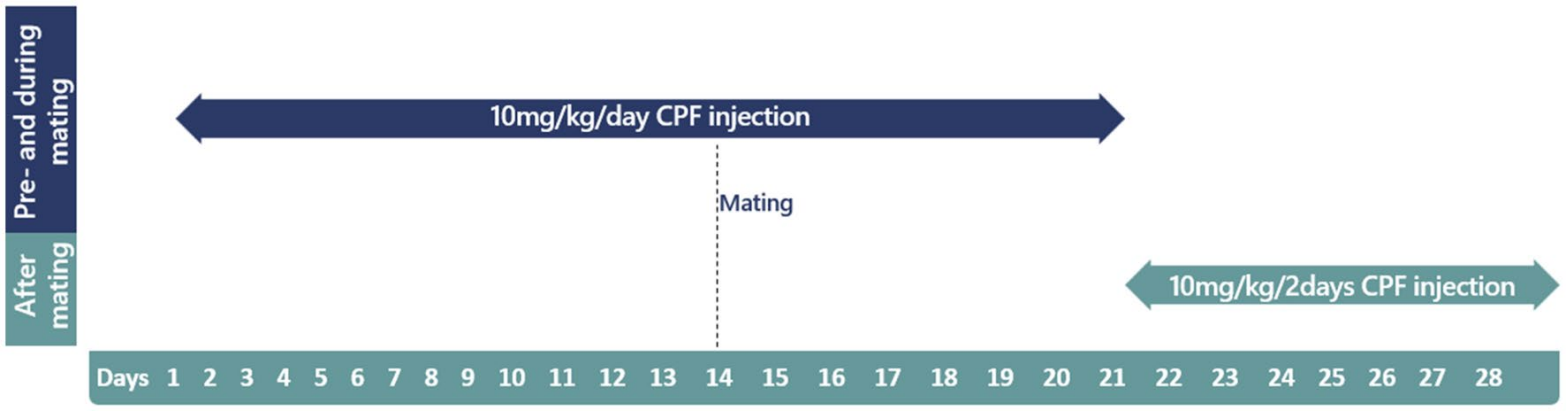
Paternal exposure to chlorpyrifos timeline. Timeline displaying chlorpyrifos exposure doses and periods of male chickens in paternal exposure. CPF: chlorpyrifos.

Chlorpyrifos administration was performed as previously described^26^. Male and female *Fayoumi* chickens (Gallus gallus domesticus) were employed as parents and were separated by sex. Eight male chicken received daily subcutaneous injections of chlorpyrifos (10mg/kg body weight, dissolved in 300 µl DMSO) at the nape of the neck for 21 days, then received maintenance injections every two days until all eggs are collected (Fig. 1). 14 days following initial treatment, the exposed males were introduced to 10 untreated females and were separated into groups as described in the chicken housing section. Eggs were collected for 10 days (maintenance exposure period), stored at 14 °C and placed in an incubator within 3 days of collection. Eggs were incubated for 20 days according to the incubator manufacturer’s instructions (50% humidity on incubation days (ID) 0–18 and 60% humidity on ID 18–20, 37.5 °C). Respective control groups received equivalent volumes of DMSO and were formed of 8 males and 10 females. Chlorpyrifos was generously supplied by Adama Ltd.

### Tissue extraction

Tissue extraction was performed as previously described^26^. Briefly, chick embryos brains were removed on incubation day 20 right before hatching. Laterally extended left intermediate medial hyperstriatum ventrale (IMHV), or intermediate medial mesopallium (IMM) corresponding to the newer nomenclature^43^ was extracted according to our modification^3,32^ of a previously described procedure^44^. The extracted IMHV tissues were stored at – 80 °C.

### RNA extraction

RNA extraction was performed as previously described^26^. Briefly, total RNA was extracted separately from the left IMHV using ISOLATE II RNA Mini Kit (Bioline, Tennessee, USA). RNA was quantified and its purity was assessed at an absorbance wavelength of 260nm using NanoDrop 2000 spectrophotometer (Thermo Fisher Scientific, Massachusetts, USA). Extracted RNA was stored at – 80 °C.

### RNA sequencing

Differential expression analysis was performed using RNA sequencing of 12 control and 12 chlorpyrifos exposed samples. The samples were separated by sex and composed of 50% males and 50% females. RNA integrity was measured using the Agilent BioAnalyzer machine (RIN was above 9). Libraries for mRNA and microRNA were prepared using KAPA Stranded mRNA Seq Kit and NEBNext Small RNA Library Prep Set, respectively, according to the manufacturers’ instructions. mRNA and small RNA libraries quantity and pooling were measured by Qubit (dsDNS HS) and quality was measured by tape station (HS). mRNA samples were sequenced using P3 flow cell and v3 P3, 50 cycle reagent kit (Illumina) on Illumina’s NextSeq2000 with 70 bp single-end reads (minimum 31 million reads per sample). microRNA samples were sequenced using NextSeq500 v2.5, 75 cycle high output kit (Illumina) on Illumina’s NextSeq500 with 50 bp single-end reads (minimum 10 million reads per sample). EdgeR package in R programming was used to identify differentially expressed genes (DEGs) in chlorpyrifos exposed offspring in comparison to control.

### Real-time qPCR analysis

Real-time qPCR analysis was carried out as previously described^45^. Complementary DNA (cDNA) was transcribed from RNA using qScript cDNA Synthesis Kit (QuantaBio, Massachusetts, USA). Expression analysis of cDNA samples was performed on the housekeeping gene: glyceraldehyde 3-phosphate dehydrogenase (GAPDH), sex-determining genes: chromodomain helicase DNA-binding Z and W (CHDZ and CHDW) and several additional genes that were selected based on mRNA sequencing results: neuronal pentraxin 2 (NPTX2), eomesodermin (EOMES), phosphoglucomutase 2 (PGM2) and catechol-o-methyltransferase domain containing 1 (COMTD1). Real-time PCR was carried out using QuantStudio 5 real-time PCR systems (Thermo Fisher Scientific, Massachusetts, USA). Genes’ availability in chicken was confirmed using the Gallus gallus genome data viewer (NCBI). Primers were selected and their specificity was verified using the Primer-BLAST tool (NCBI). The primers were then synthesized commercially (Merck, Israel). Primers used and their sequences are listed in Table 1. Genes amplification was performed using PerfeCTa SYBR Green FastMix, ROX (QuantaBio, Massachusetts, USA) under the following conditions: preincubation at 95 °C for 20 s, followed by 40 cycles of denaturation at 95 °C for 3 s and annealing at 60 °C for 30 s. Relative quantifications of target genes were normalized to GAPDH levels (coefficient of variation of GAPDH = 0.006), and calculated using the 2^−ΔΔCt^ method, as previously described^46^. RT-PCR product specificity was confirmed using melt curve analysis. Compared to RNA sequencing, qPCR analysis was performed on a larger number of animals using samples different from those used in sequencing.

**Table 1:** Sequences of the used primers.

Primer	Sequence (5′–3′)
NPTX2 forward	CTTCATAATGAGACATCAGCC
NPTX2 reverse	GAAGGGACACTTTGAACTC
EOMES forward	GTGGAAGTGACTGAAGATG
EOMES reverse	TGAGCTGAGTAATATCGGTG
PGM2 forward	CAGACAACTCAGGGATTTTG
PGM2 reverse	ACGAGCATCAAATCCAATC
COMTD1 forward	TGCTTGGAGATGTACTATGGGT
COMTD1 reverse	TGGAAGTCAGTCATTCTACACACA
CHDZ forward	AGTCAGGCAGTCAATCCGAA
CHDZ reverse	CCACTGTCTGATGATGCTGC
CHDW forward	GGGATTCTGAGTGAAGACTGG
CHDW reverse	CTGTCCTGTGCCCTTCTTG
GAPDH forward	TGGAGCCCCTGCTCTTCA
GAPDH reverse	GGAACAGAACTGGCCTCTCACT

### Sex determination

CHDW and CHDZ primers enabled the discrimination between male and female offspring. This differentiation verified the results of the visual inspection of the gonads.

### Gene expression analysis

Reads from RNA and small RNA sequencing were aligned to the chicken genome (galGal6). Raw count data were used for differential expression analysis using edgeR package in R programing^47^. Genes in mRNA and small RNA sequencing analyses with false discovery rate (FDR) < 0.1 (calculated by edgeR package) were considered statistically significant. Cut off value of FDR < 0.1 is common in the literature as is shown in leading studies, a few examples are quoted below^48–52^. No fold change value was set as a cut off in the DEGs. Analyzed data were displayed using volcano plot and heatmap. Heatmap was plotted by http://www.bioinformatics.com.cn/srplot (accessed on 24 August 2023), an online platform for data analysis and visualization.

### qPCR statistical data analysis

Multiple level analysis of variance (ANOVA) was employed for comparing groups as previously described^26^. Data were expressed as Mean ± SEM. Genes expression in the offspring paternally exposed to chlorpyrifos was compared to the control offspring that were not exposed to chlorpyrifos nor were their parents. One-way ANOVA was used to test the effects of treatment on chlorpyrifos exposed and control groups where the dependent variable was gene expression score. Preliminary, global ANOVA clearly showed no sex effect or interaction. Subsequently, female and male offspring scores were pooled for the analysis. Post-hoc Tukey’s test was used when appropriate. Significance level for all employed tests was considered at p < 0.05.

### Graphical data presentations

#### Volcano plot

A scatter plot that displays unstandardized signal (log fold change) against standardized signal (− log_10_ (p-value))^53^. Log fold change (logFC) represents the log fold change of the genes in chlorpyrifos exposed samples compared to control samples, with values above 0 indicating increased gene expression and values under zero indicating decreased gene expression.

#### Heatmaps and hierarchical clustering

Used in expression analysis studies for quality control and data visualization^54^. The z-score (sample value – mean/standard deviation) is represented in colors, with the dark blue representing the least z score and dark red representing the highest score. Hierarchical clustering puts together similar samples into groups called clusters and is represented by a dendrogram which indicates similarity and order of each formed cluster.

### Gene set and protein–protein interaction enrichment analyses

Gene Ontology (GO) molecular function and Kyoto Encyclopedia of Genes and Genome (KEGG) analyses^55–57^ were performed via Metascape (version 3.5)^58^. Enriched pathways network was visualized using Cytoscape (version 3.9.1)^59^. Cell type signatures^60^, disease associated genes (DisGeNET^61^) and transcription factor analyses were also performed on the DEGs using the Metascape platform. FDR < 0.1 was used to select the analyzed genes. Protein–protein interaction enrichment analysis was generated using Metascape^58^ and was based on several databases including STRING^62^, BioGrid^63^, OmniPath and InWeb_IM^64^. Only physical interactions in STRING (physical score > 0.132) and BioGrid were used. Molecular complex detection (MCODE^65^) algorithm was applied to identify densely connected network components. Protein–protein interaction enrichment analysis was performed on DEGs with FDR < 0.1 in pooled females and males samples.

## Results

### mRNA sequencing

**Figure 2:**
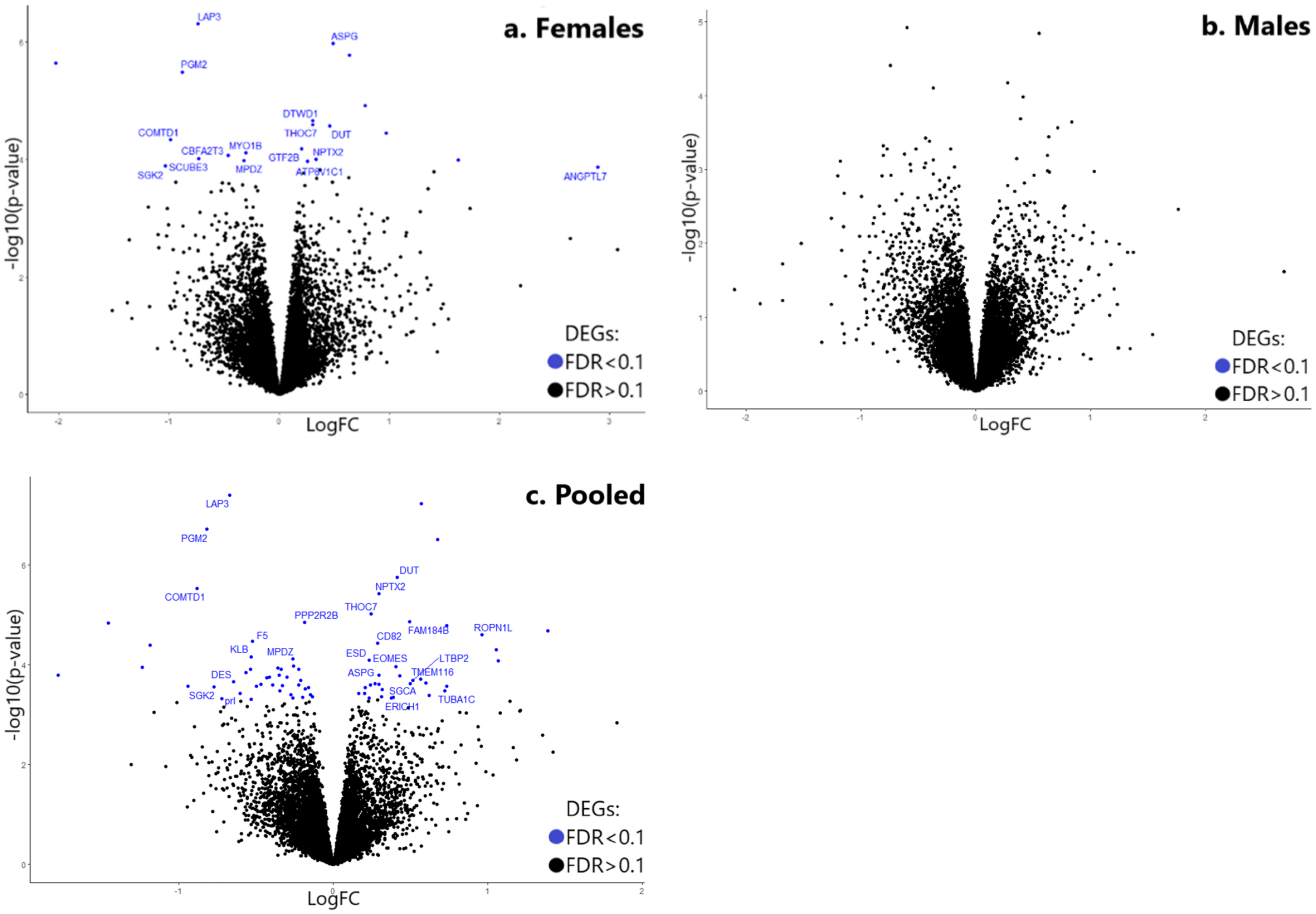
Volcano plots showing differentially expressed genes in female offspring (**a**), male offspring (**b**) or pooled female and male offspring (**c**). Volcano plot presenting DEGs with FDR < 0.1 (blue) or FDR > 0.1 (black). p values were calculated by edgeR. p-value is represented in the “x” axis. Log fold change (logFC) is presented by the “y” axis where values above 0 indicate positive log fold change and values under zero indicate negative log fold change.

**Figure 3:**
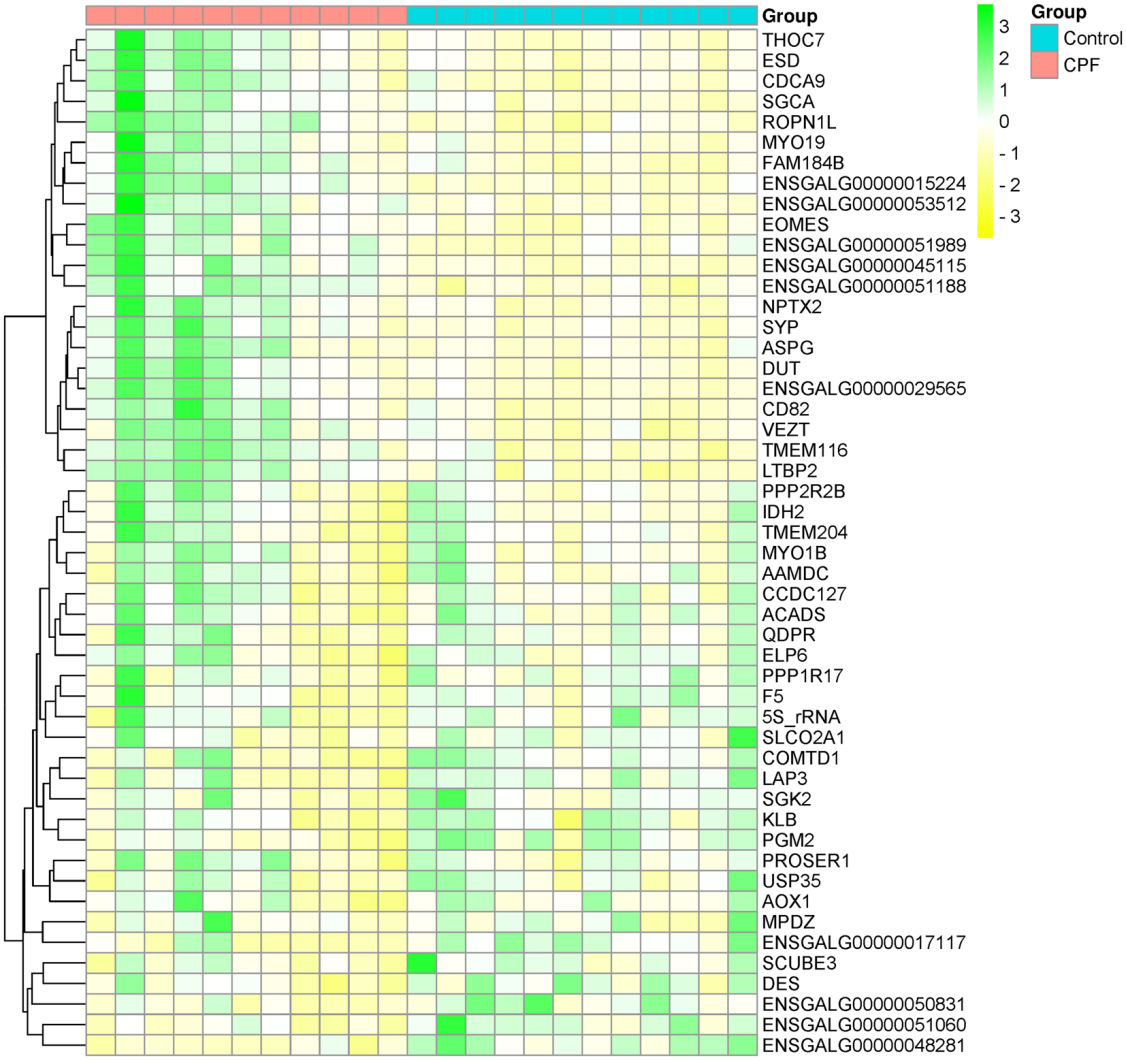
Heatmap of the 50 most significant differentially expressed genes from pooled samples. Heatmap of the 50 most significant DEGs with FDR < 0.1. The color corresponds to the scaled expression levels (yellow—negative, green—positive). Genes are displayed as gene names or Ensemble IDs (if gene name is not identified). Samples are divided into control (blue) and chlorpyrifos exposed (pink) samples at the top of the heatmap. Dendrogram in the left side of the heatmap represents the hierarchical clustering. *CPF* chlorpyrifos. Heatmap was plotted online using the website: http://www.bioinformatics.com.cn/srplot.

**Figure 4:**
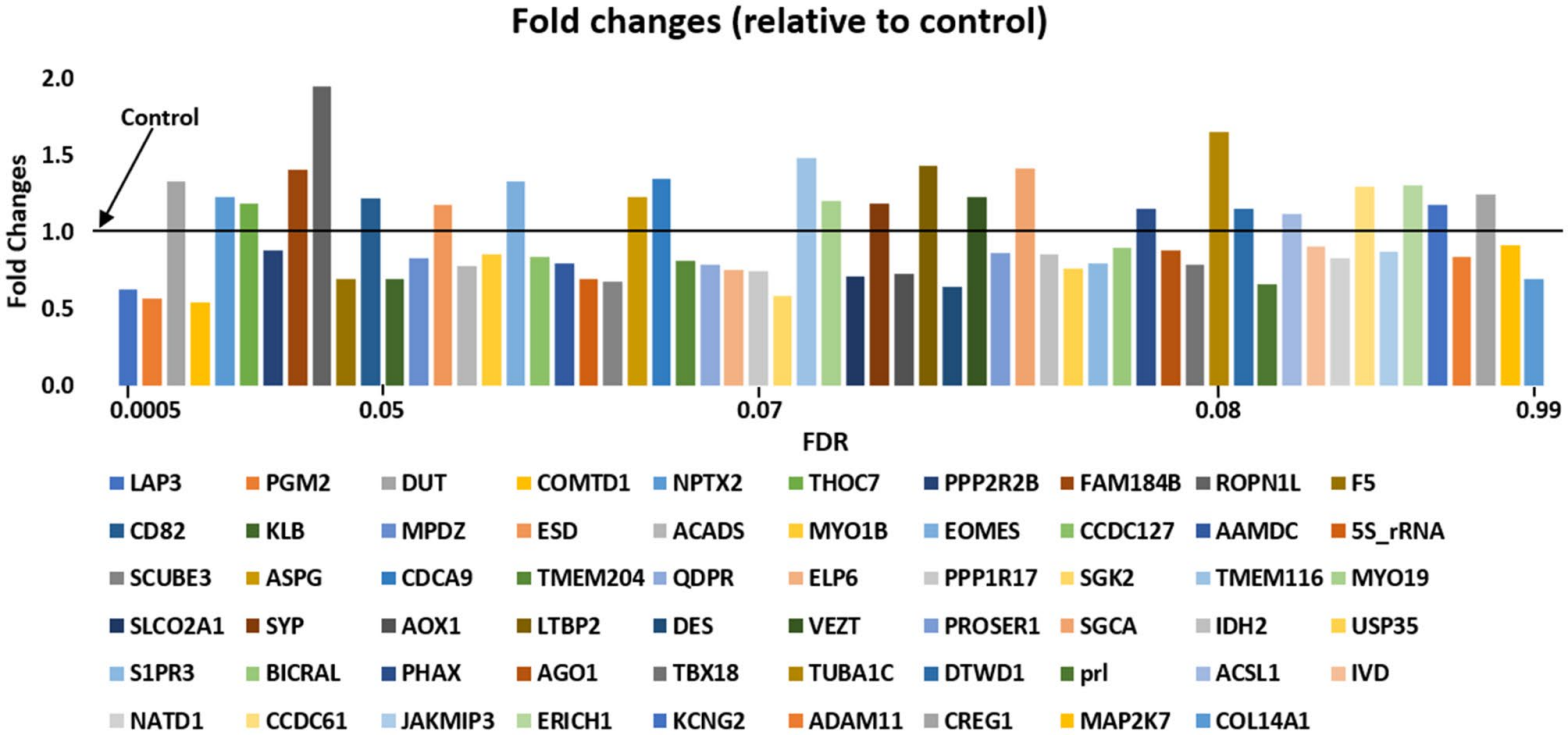
Fold changes of the differentially expressed genes (FDR < 0.1) in the offspring paternally exposed to chlorpyrifos (pooled samples). DEGs fold changes from control in the offspring paternally exposed to chlorpyrifos. Each gene is presented in different color as indicated under the graph. The order of the genes in the graph (from left to right) is the same order of the genes’ names presented under the graph (from left to right) starting with LAP3 (dark blue) and ending with COL14A1 (light blue). Relative FDR values increase from left to right starting with LAP3 (presenting lowest FDR value) and ending with COL14A1 (presenting highest FDR value). Only genes with identified names were displayed.

**Figure 5:**
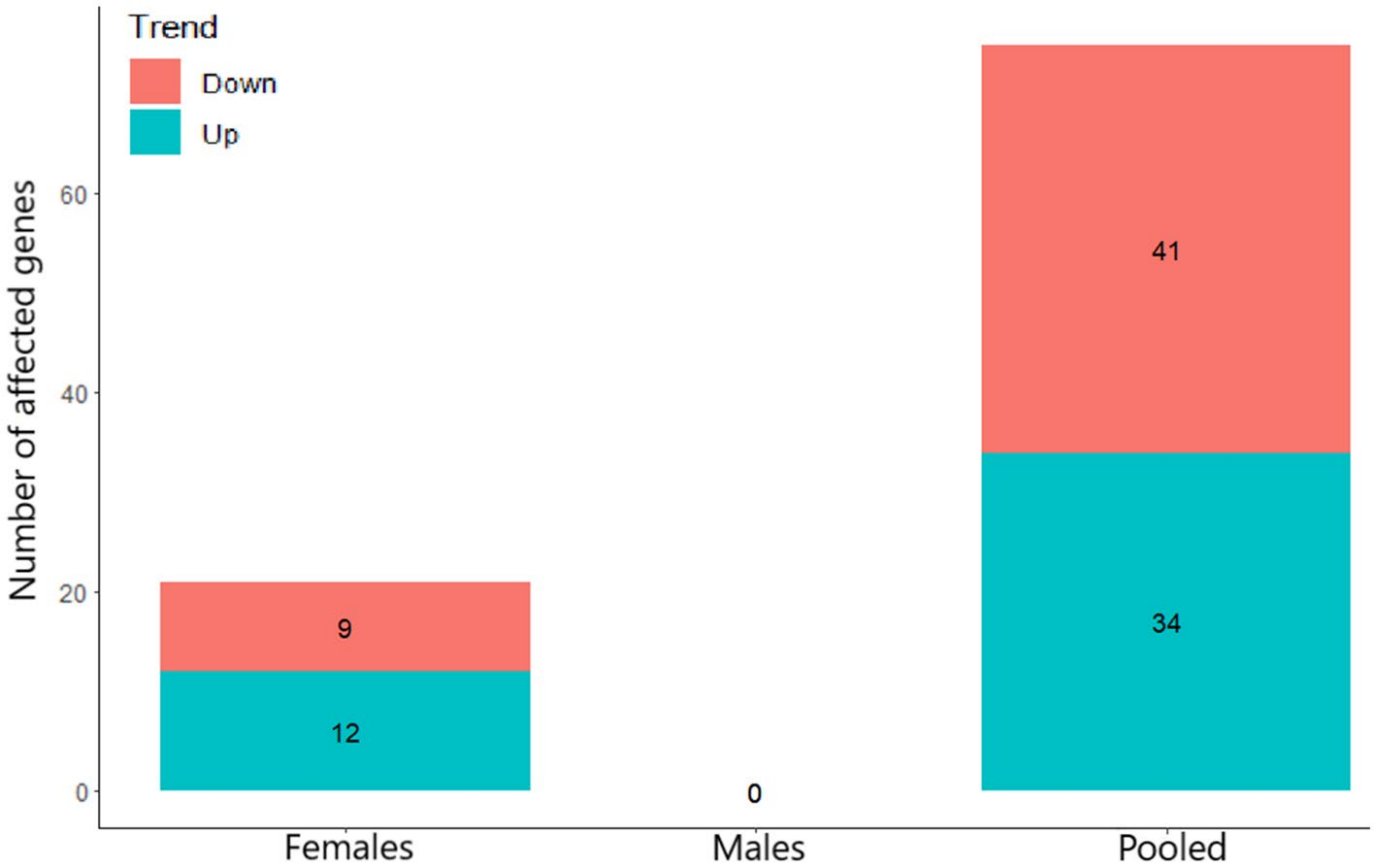
Differentially expressed genes (FDR < 0.1) trend in female offspring, male offspring and pooled female and male offspring. Number of DEGs with FDR < 0.1 is presented in the Y axis. The trend represents upregulation (Up) and downregulation (Down) of the DEGs in females, males and pooled females and males offspring. The number of upregulated and downregulated genes is presented inside each column.

At significance cut-offs corresponding to FDR < 0.1, 75 differentially expressed genes were identified (Figs. 2, 3, 4, 5). Volcano plots of the significantly changed genes comparing control and chlorpyrifos exposed samples in female and male offspring as separate, and in pooled females and males are shown in Fig. 2.

The results suggest marked changes in the expression of numerous genes (in pooled samples) after paternal exposure to chlorpyrifos. DEGs were more pronounced in female offspring (Fig. 2a) compared to male offspring (Fig. 2b).

The heatmap of the 50 most significant differentially expressed genes are shown in Fig. 3. Samples are divided into chlorpyrifos exposed (pink) and control (blue) samples and are shown at the top of the heatmap.

The heatmap displayed different sets of genes with similar patterns, primarily “THOC7, ESD, CDCA9, SGCA, ROPN1L, MYO19 and FAM184B” set which showed increased genes expression in the chlorpyrifos exposed samples compared to control.

Fold changes of the differentially expressed genes relative to control are presented in Fig. 4. Each gene was labelled in a different color. The FDR value of the displayed genes increases (significance decreases) from left to the right.

Fold changes analysis showed positive and negative DEGs fold changes seen as columns above and below 1, respectively. Of the total 75 DEGs, only genes with known names were displayed while genes showing differentially expressed alterations with no known names (seen as gene Ensembl in sequencing data) were not displayed.

The number of the differentially expressed genes (FDR < 0.1) obtained in female and male offspring as separate and as pooled female and male offspring is indicated in Fig. 5.

Figure 5 suggests that gene expression changes in the female offspring after paternal exposure to chlorpyrifos were more pronounced than the changes observed in the male offspring.

### Small RNA sequencing

**Figure 6:**
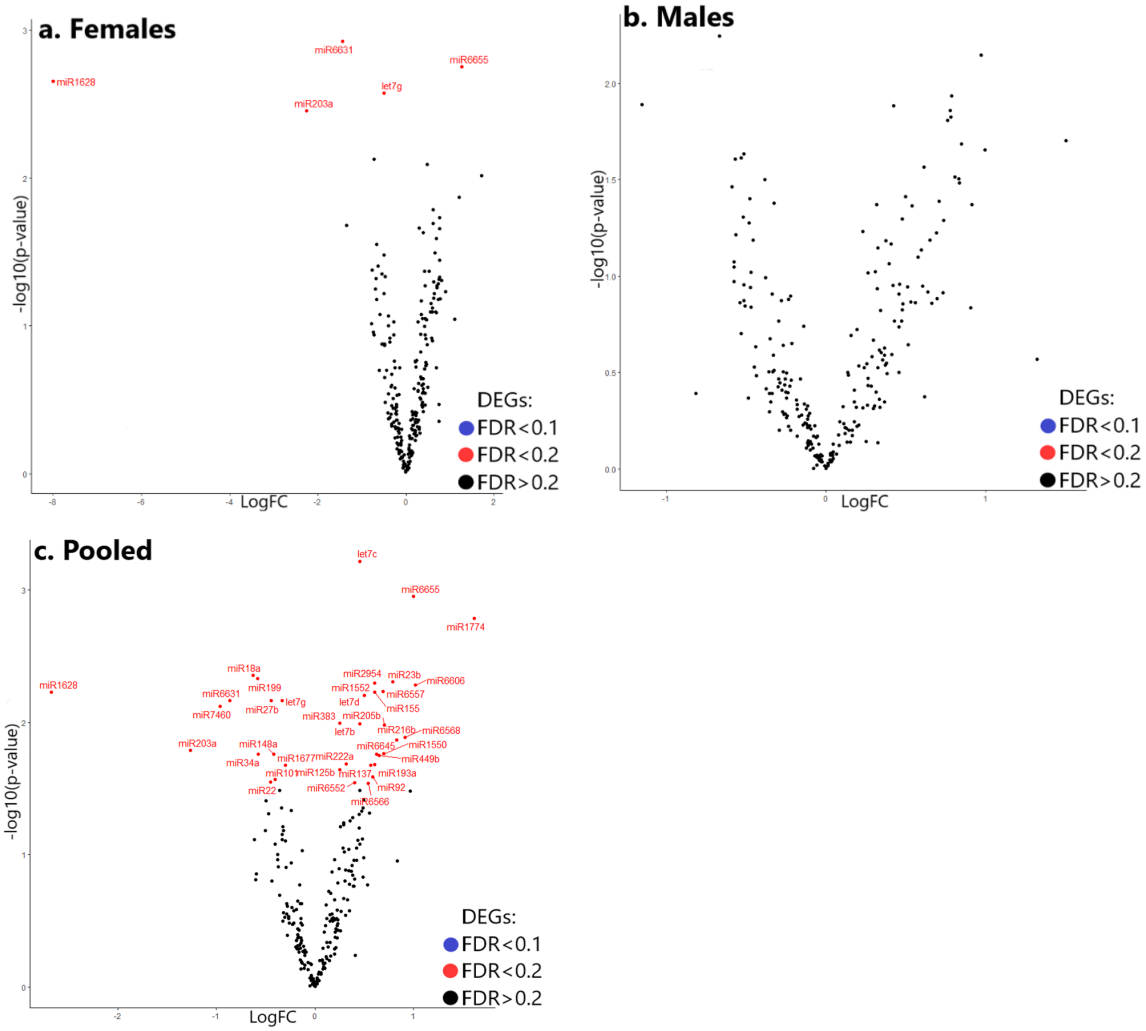
Volcano plots showing differentially expressed microRNA genes in female offspring (**a**), male offspring (**b**) or pooled female and male offspring (**c**). Volcano plot presenting DEGs with FDR < 0.1 (blue), FDR < 0.2 (red; non-significant) or FDR > 0.2 (black; non-significant). p values were calculated by edgeR. p-value is represented in the “x” axis. Log fold change (logFC) is presented by the “y” axis where values above 0 indicate positive log fold change and values under zero indicate negative log fold change.

None of the investigated microRNA genes were significantly altered (FDR < 0.1) as proposed by small RNA sequencing analysis. Consequently, results other than the volcano plots (Fig. 6) were not displayed.

### Gene set enrichment analysis

**Figure 7:**
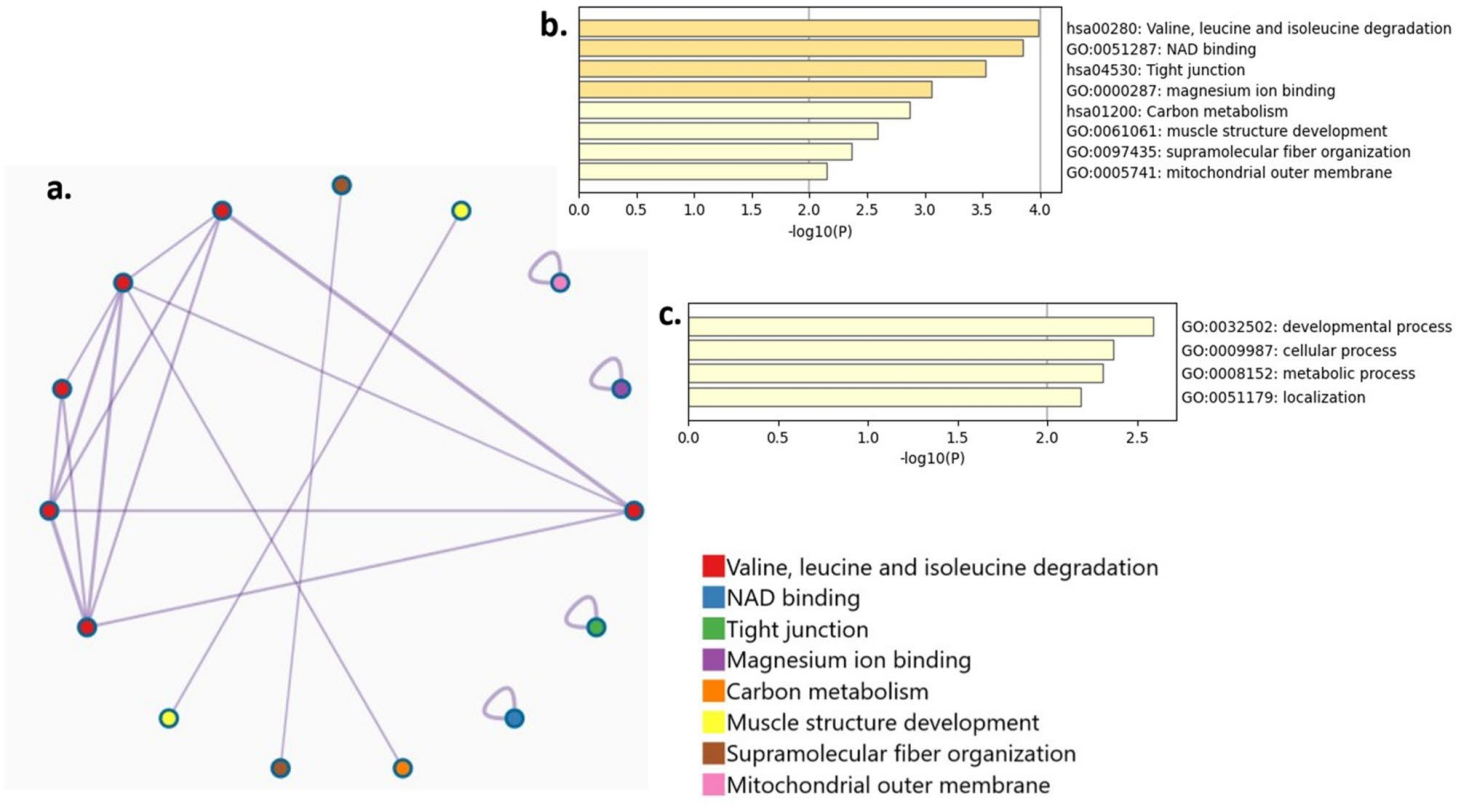
Gene set enrichment analyses showing enriched pathways network (**a**) their significance (**b**) and top-level KEGG pathways and GO biological processes (**c**) of the differentially expressed genes. Gene sets representing similar pathways were clustered and labelled with a specific color as indicated in the tiles. Significance is represented by − log10(p) with pathways having − log10(p) values ≥ 3 marked in yellow. Pathways hsa00280, hsa04530 and hsa01200 in top level KEGG pathways and GO biological processes (**b**) were identified by KEGG^55–57^.

**Figure 8:**
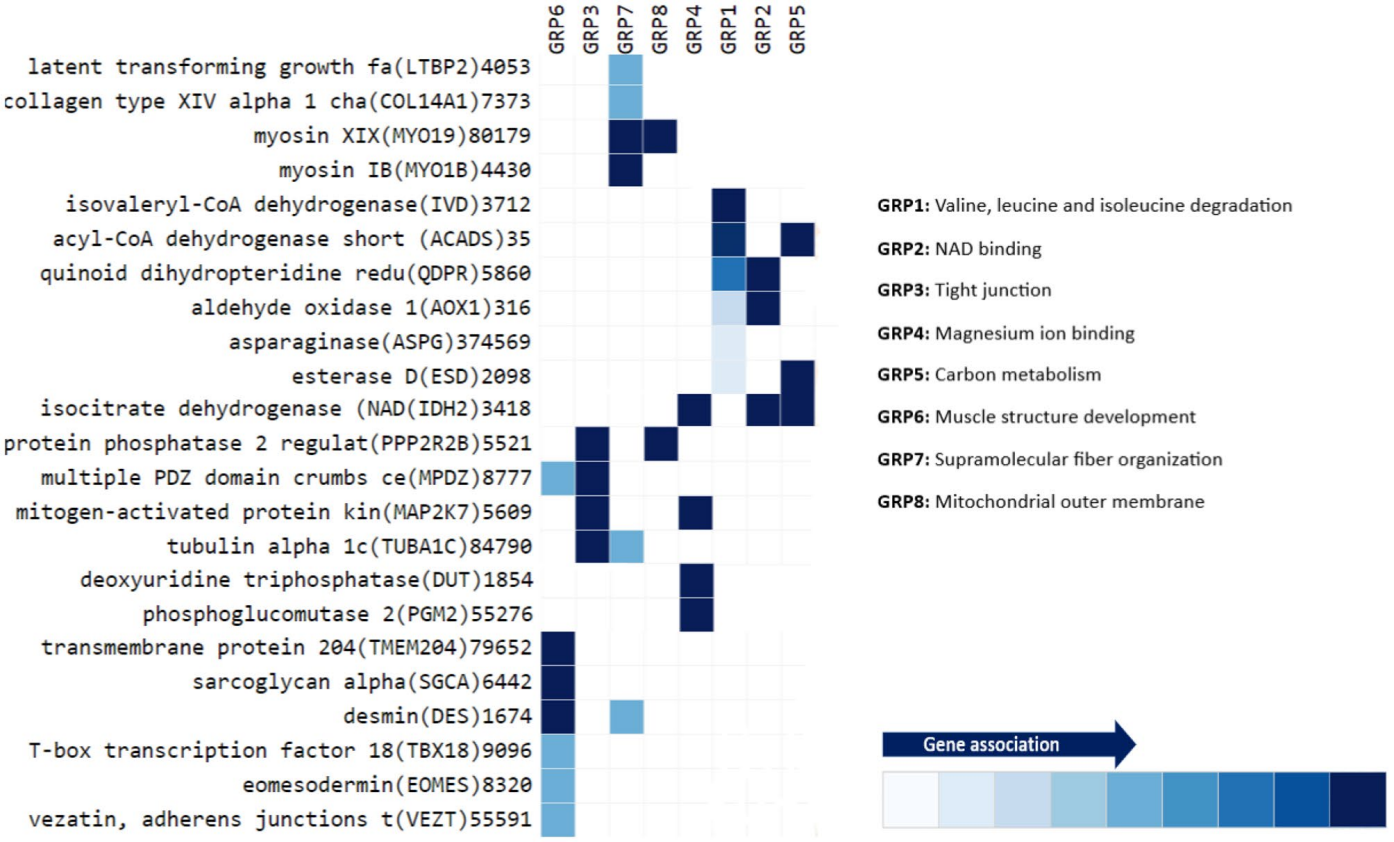
Enriched pathways members heatmap. The involvement of each gene in the GO pathway is represented by the tile color with dark blue indicating strong association and light blue indicating weak association. Each pathway was presented as a group (GRP) in the heatmap, and the name of each group was indicated on the right side of the figure. Enriched pathways members heatmap was plotted via Metascape (version 3.5) using the website: https://www.metascape.org. GRP1, GRP3 and GRP5 were identified by KEGG^55–57^.

**Figure 9:**
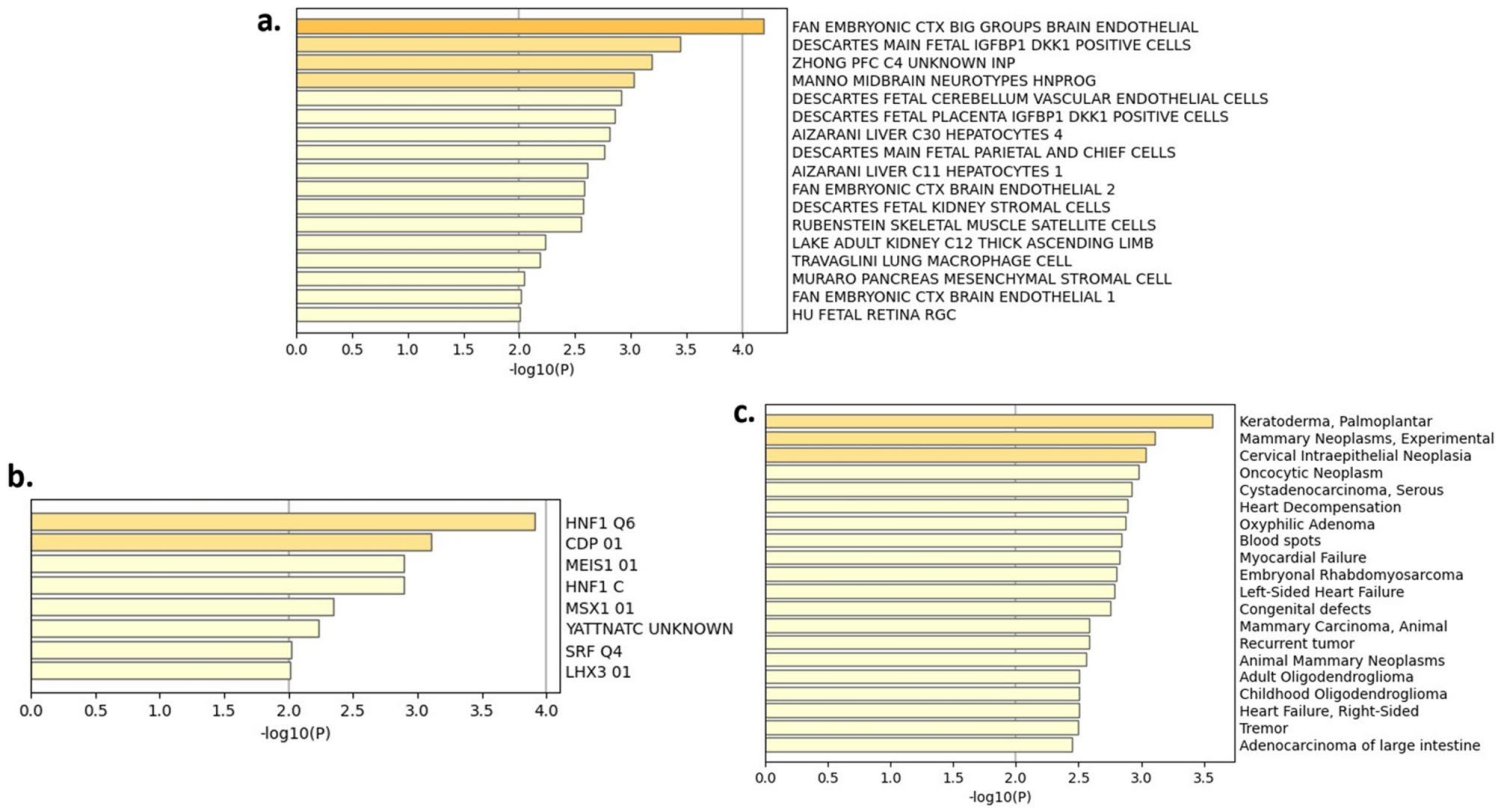
Cell type signatures (**a**), transcription factor analyses (**b**) and disease associated genes (**c**) of the differentially expressed genes. Significance is represented by − log10(p) with cells/genes/diseases having − log10(p) values ≥ 3 marked in yellow.

Based on the results obtained from mRNA sequencing, gene set enrichment analysis was used to identify gene sets possessing similar/related pathways and relate those sets to biological functions, diseases and transcription factors (Figs. 7, 8, 9). Eight major biological pathways were identified (Fig. 7) including valine, leucine and isoleucine degradation, nicotinamide adenine dinucleotide (NAD) binding, tight junction, magnesium ion binding, carbon metabolism, muscle structure development, supramolecular fiber organization and mitochondrial outer membrane.

Heatmap of the gene members of enriched pathways **(**Fig. 8**)** proposed that several genes are involved in more than one biological pathway, especially isocitrate dehydrogenase which was involved in magnesium ion binding, NAD binding and carbon metabolism pathways.

Cell type signatures, disease associated genes and transcription factor analyses (Fig. 9) mainly proposed the involvement of embryonic brain, palmoplantar keratoderma and hepatocyte nuclear factor 1 (HNF1) in the analyzed DEGs, respectively.

### Protein–protein interaction enrichment analysis

**Figure 10:**
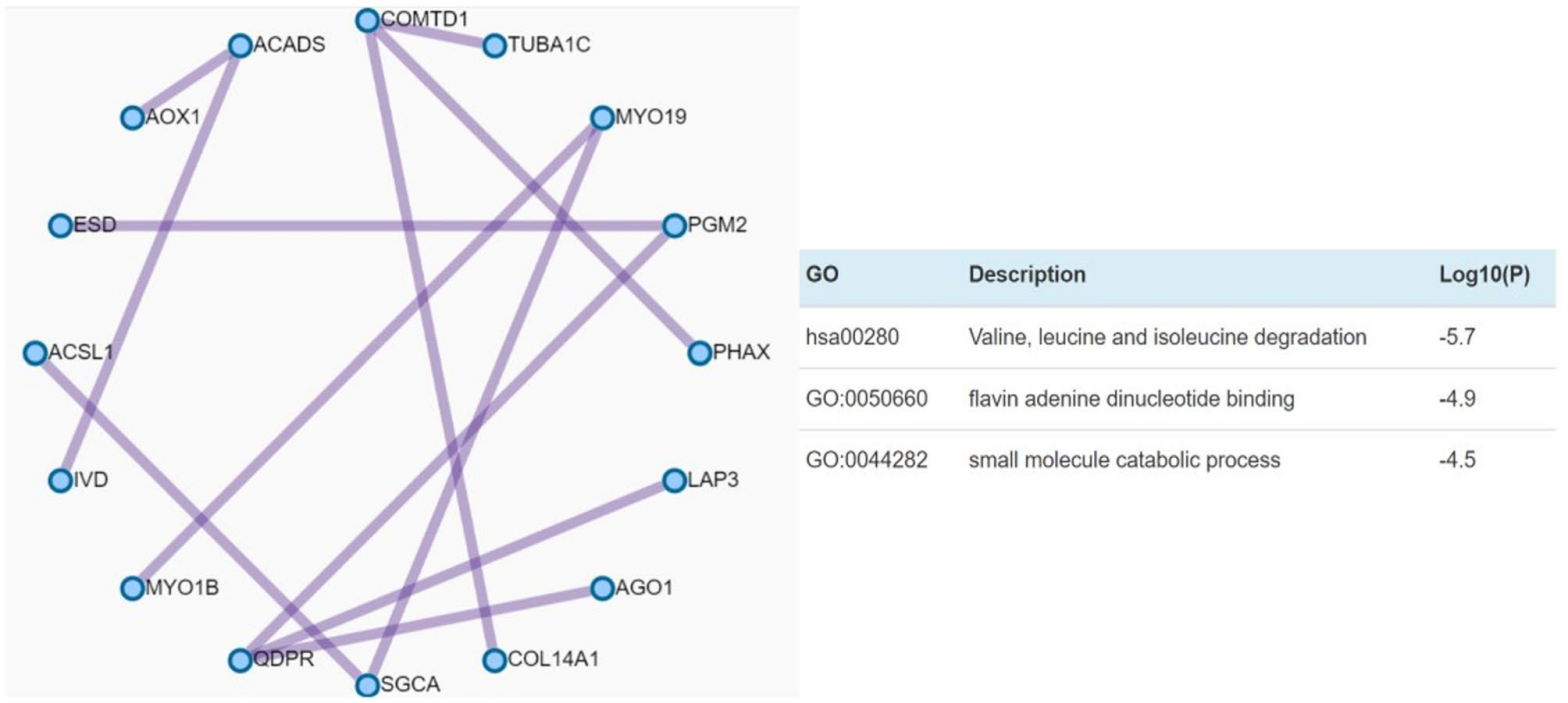
Protein–protein interactions of the differentially expressed genes. Drawn lines represent interactions between the linked proteins. The three most involved (best scoring) pathways in the protein–protein interaction analysis, as identified by MCODE, are shown on the right side of the graph.

Protein–protein interaction enrichment analysis integrates publicly available sources of protein–protein interaction data. Several protein–protein interactions of the differentially expressed genes were identified (Fig. 10), where COMTD1 and quinoid dihydropteridine reductase (QDPR) showed the most interactions. COMTD1 interacted with tubulin alpha 1C (TUBA1C), phosphorylated adaptor for RNA export (PHAX) and collagen type XIV alpha 1 chain (COL14A1) while QDPR interacted with argonaute RISC component 1 (AGO1), LAP3 and PGM2. The main biological pathways involved in the protein–protein interaction included valine, leucine and isoleucine degradation, flavin adenine dinucleotide binding and small molecule catabolic process.

### qPCR analysis

**Figure 11:**
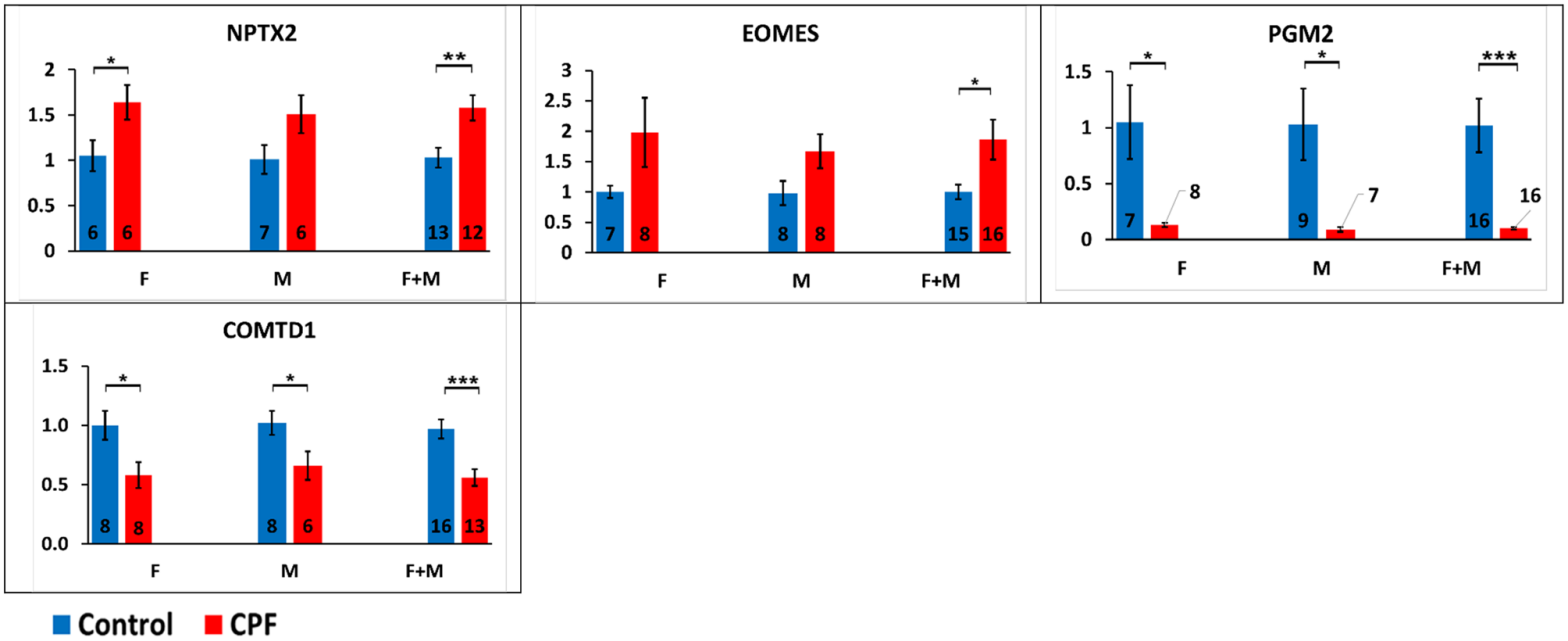
qPCR gene expression analysis of NPTX2, EOMES, PGM2 and COMTD1 genes in the offspring after paternal exposure to chlorpyrifos. Relative gene expression results presented as Mean ± SEM. Number of samples (n) is presented inside each column (except for PGM2-CPF samples). *M* male offspring, *F* female offspring. *p < 0.05, **p < 0.005, ***p < 0.001. *NPTX2* pentraxin 2, *EOMES* eomesodermin, *PGM2* phosphoglucomutase 2, *COMTD1* catechol-o-methyltransferase domain containing 1.

qPCR analysis was used to confirm gene expression observations obtained in mRNA sequencing on a larger number of animals using samples different from those used in sequencing. Preliminary, global ANOVA clearly showed no sex effect (F[1,115] = 0.18, p = 0.67) or treatment by sex interaction (F[3,113] = 0.67, p = 0.57). Subsequently, the scores of the female and male offspring were pooled for the analysis. In the successive treatment analysis, there have been no significant treatment effect because the genes were altered to different directions (F[1,115] = 1.60, p = 0.21). Therefore, there was a robust gene effect (F[3,113] = 13.97, p < 0.0001) and an obvious treatment by gene interaction (F[7,109] = 15.26, p < 0.0001). Separated analysis for each gene showed that paternal exposure to chlorpyrifos increased NPTX2 (females: F[1,10] = 5.35, p < 0.05; males: F[1,11] = 3.78, p = 0.08; pooled: F[1,23] = 9.75, p < 0.005) and EOMES (females: F[1,13] = 2.52, p = 0.14; males: F[1,14] = 4.01, p = 0.06; pooled: F[1,29] = 5.86, p < 0.05) genes expression and decreased PGM2 (females: F[1,13]  = 8.92, p < 0.05; males: F[1,14] = 6.51, p < 0.05; pooled: F[1,30] = 14.43, p < 0.001) and COMTD1 (females: F[1,14] = 6.87, p < 0.05; males: F[1,12] = 5.43, p < 0.05; pooled: F[1,27] = 14.57, p < 0.001) genes expression in the offspring. Gene expression differences in each gene (NPTX2, EOMES, PGM2 and COMTD1) are shown in Fig. 11. qPCR gene expression analysis results in NPTX2, EOMES, PGM2 and COMTD1 were parallel to those obtained in mRNA sequencing.

## Discussion

Our earlier studies demonstrate that pre-hatch exposure to neuroteratogens affects the offspring’s neurogenesis, neurotransmission, learning and memory^3,32^. In an attempt to expand those findings in a paternal model, we performed whole-transcriptome analysis on the offspring paternally exposed to the developmental insult (chlorpyrifos) to provide a comprehensive evaluation of gene expression changes. This is a pioneering study that utilizes next generation sequencing to investigate the effects of paternal exposure to developmental insults in a novel avian model.

We have previously shown that pre-hatch exposure to chlorpyrifos affects the offspring’s learning and memory as a result of changes in neurogenesis and neurotransmission related genes expression and the abolishment of activation/translocation of PKC isoforms [PKC isoforms were not activated/translocated after introducing the IMHV of chlorpyrifos exposed offspring to carbachol (cholinergic agonist)]^3,14^.

In the current study, we hypothesized that paternal exposure to the developmental insult would have extensive effects on the offspring’s gene expression, especially neurogenesis and neurotransmission related genes pertaining to deficits in learning and memory as established in our previous studies on the pre-hatch model^3,32^. Indeed, the obtained results suggest that the expression of several neurogenesis, neurotransmission, learning and memory related genes was affected in the offspring after paternal exposure to the developmental insult. Those genes were either downregulated: protein phosphatase 2 regulatory subunit B beta (PPP2R2B), sphingosine-1-phosphate receptor 3 (S1PR3), ADAM metallopeptidase domain 11 (ADAM11) and mitogen-activated protein kinase kinase 7 (MAP2K7); or upregulated: CD82 molecule (CD82), synaptophysin (SYP), NPTX2.

Gene expression changes in PPP2R2B were previously linked to working memory performance in the prefrontal cortex^66^. The authors claimed that protein phosphatase 2A (PP2A), the phosphatase that is regulated by PPP2R2B, has a crucial role in neurotransmission (through its involvement in D2 cAMP—independent signalling) and in cognition in the prefrontal cortex^66^.

Previous studies performed on young adult mice report that CD82 overexpression in the hippocampus impairs learning, memory and synaptic plasticity, whereas CD82^-/-^ mice show hippocampus-related memory functions improvement^67^.

Synaptophysin is considered a crucial molecule in synaptogenesis and is necessary for some forms of learning and memory^68^. Chen et al. showed that densities of synaptophysin immunostaining significantly decreased in the hippocampal formation area in an immobilized mice (sedentary) group compared to younger mice, whereas a significant increase in synaptophysin densities was observed in trained mice compared to their age-matched sedentary controls. The authors claim that the increased expression of synaptophysin may be associated with anatomical sprouting and synaptogenesis^68^.

Ablation of S1PR3 was shown to impair spatial working memory and neuron excitability in the hippocampus^69^. Increased interspike intervals and decreased input resistance were observed in principal neurons in CA3 area lacking S1PR3^69^.

ADAM11 deficient mice showed deficits in spatial learning and motor coordination as observed in hidden water maze and rotating rod task skills^70^.

Deficits in prefrontal cortex-dependent working memory and cognitive impairments were observed in MAP2K7^+/−^ mice mainly seen as impaired attention, a vigilance decrement deficit and unstable cognitive processing in an attentional task^71,72^.

NPTX2 is a member of the neuronal pentraxin family which regulate synaptogenesis and glutamate signaling by clustering AMPARs and contribute to multiple forms of developmental and adult synaptic plasticity^73–77^. Pekley et al. showed that pentraxins coordinate circuit integration of parvalbumin interneurons and excitatory synapse maturation^73^. Early postnatal NPTX2^−/−^/NPTXR^−/−^ mice were shown to have delayed circuit maturation with a prolonged critical period permissive for giant depolarizing potentials, whereas juvenile NPTX2^−/−^/NPTXR^−/−^ mice showed reduced feedforward inhibition generating a circuit deficient in rhythmogenesis^73^.

In addition, changes in epigenetic genes including EOMES and COMTD1 were detected. EOMES is considered a transcription factor that regulates neurogenesis^78^, while COMTD1 encodes an effective methyltransferase with O-methyltransferase activity^79–81^. It is also predicted to enable S-adenosylmethionine (SAMe) dependent methyltransferase activity^82^.

The effect of paternal exposure to the developmental insult on neurogenesis was further confirmed by gene list enrichment analysis which identified NAD binding as a major biological pathway involved in the DEGs. NAD has been previously linked to neurogenesis through its action on brain derived neurotrophic factor (BDNF) gene expression^83^.

Small RNA sequencing results proposed no significant expression changes in microRNA genes after paternal exposure to the developmental insult. This possibly indicate that microRNA is not the major epigenetic regulator mediating mRNA genes expression changes in the offspring after paternal exposure.

It is important to note that this is a neurobehavioral teratology study which by definition investigate small effects^84^, which means that using a more conservative FDR value (< 0.05) may cause some of the results to be lost (type II error). Review of the literature clearly demonstrate that this is a common FDR cut off value (< 0.1) as is shown in numerous leading studies, here we cited several examples^48–52^. In addition, the results of some of the genes in the present study were replicated and confirmed using qPCR analysis.

qPCR analysis was performed on NPTX2, EOMES, PGM2 and COMTD1 genes in order to validate the results obtained in mRNA sequencing. Most analyzed genes were selected based on their significance value (FDR < 0.01) in mRNA sequencing. qPCR Gene expression changes in NPTX2, EOMES, PGM2 and COMTD1 genes were similar to the changes obtained in mRNA sequencing.

The spermatogenesis period is around 14 days in avian models^85–88^, so in order to affect the fathers’ germ cells they were exposed to the developmental insult for two weeks prior to mating with unexposed females.

Sex effect is a well-established factor in neurobehavioral teratology, and has been previously shown, in previous studies including our own, to affect gene expression in the offspring (different effect in male and female offspring) after prenatal or parental preconception exposure to the neuroteratogens heroin and chlorpyrifos, respectively^24,26,89^. Consequently, we decided to separate between female and male offspring in the current study. Indeed, female offspring were apparently more affected by paternal exposure to the developmental insult than males. This was suggested by volcano plot (Fig. 2) where significant DEGs (FDR < 0.1) possessing both upregulation and downregulation trends (Fig. 5) were only detected in the female offspring.

IMHV (mainly left IMHV) is considered crucial for memory and learning in avian models^90,91^ and because exposure to the developmental insult affects these phenotypes^3^, the left IMHV was selected for mRNA extraction and analysis in the current study.

In order to reduce possible confounding factors in the offspring (hatching day, intake of food, etc.) and perform a study with minimal bias, all embryos were sacrificed one day prior to hatching (ID20).

Future studies should correlate the observed expression changes in non-neuronal related genes (genes that are not related to neurogenesis, neurotransmission, learning or memory) to the deficits that could occur after early exposure to developmental insults^3,92–95^.

The present research strongly suggests that paternal exposure to the developmental insult, chlorpyrifos markedly affects the offspring’s genes expression and points to the most pronounced of them. Further studies on functional deficits emphasizing the differences in female and male offspring, should tie the molecular alterations observed and the expected neurobehavioral or other deficits in the offspring (as shown in previous studies including our own^3^) in causal relationships which would provide the groundwork for the reversal of these deficits (as we previously established in the pre-hatch model^32^), towards future studies for clinical application.

## Data Availability

The datasets generated and/or analyzed during the current study are available in the [NCBI] repository [PRJNA953674] via the link: https://www.ncbi.nlm.nih.gov/Traces/study/?acc=PRJNA953674&o=acc_s%3Aa.
